# Cases of Scirrhus of the Breast

**Published:** 1830-01

**Authors:** Herbert Mayo

**Affiliations:** Surgeon to the Middlesex Hospital, &c.


### THE LONDON
### Medical and Physical Journal.

N° 371, VOL. LXIII.] JANUARY 1830. [N° 43, *New Series.*

For many fortunate discoveries in medicine, and for the detection of numerous errors, the world is indebted to the rapid circulation of Monthly Journals; and there never existed any work, to which the Faculty, in Europe and America, were under deeper obligations than to the *Medical and Physical Journal of London*, now forming a long but an invaluable series.—*Rush.*

### ORIGINAL PAPERS, AND CASES,

### OBTAINED FROM PUBLIC INSTITUTIONS AND OTHER AUTHENTIC SOURCES.

### SCIRRHUS OF THE BREAST.

*Cases of Scirrhus of the Breast.* By HERBERT MAYO, F.R.S. Surgeon to the Middlesex Hospital, &c.

THE following cases exemplify different forms of a complaint too frequent not to have been repeatedly witnessed by all the readers of this Journal. They are instances, 1, of simple scirrhus; 2, of scirrhus assuming a fungoïd character: under a third head, I have described a tumor which, although probably not malignant, more nearly resembles scirrhus than any other morbid structure.

I.

A woman, about forty-five years of age, had been afflicted several months with scirrhus of the mammary glands and of the integuments. The right breast was nearly flat, the nipple retracted; the integument immediately covering the gland, as well as the adjacent integument upon the right side and front of the body, was thickly studded with hard nodules. These nodules were a little elevated; most of them were about a third of an inch in diameter, one or two an inch, one an inch and a half; the largest among them were covered with a crust or thick scab. There were several lymphatic glands in the axilla enlarged and indurated; the right arm was œdematous and greatly swollen.

The œdema was temporarily diminished, as I have seen happen in other similar cases, by the application of a blister to the swollen arm. The patient complained of darting pains and a sense of burning in the right breast. The left breast was harder than natural, and the surface of the gland felt coarsely granulated; the nipple was not retracted.

Upon the death of this patient, I carefully examined the body, and remarked the following appearances:

The gland of the right breast, when divided, was of a grey colour, and presented a texture like that of an unripe pear, *cutting* with a characteristic resistance and sound. Tubuli lactiferi were seen upon the surface of the section, both in the gland and nipple, containing a thick white secretion. The right pectoral muscle, to which the breast strongly adhered, had two or three large, firm, white tubercles in its substance.

The structure of the left breast was different from that of the right: the gland was greatly hardened, but had preserved its natural colour; its surface was exceedingly irregular, being raised into innumerable little knots; and, when divided, the section had a corresponding character, and bore some resemblance to the section of an indurated pancreas. The left breast was readily separable from a thick layer of cellular membrane, which intervened between it and the pectoral muscle: this layer of membrane was studded with numerous small, white, hard, flattened nodules, varying from two to three lines in diameter.

The lymphatic glands that were affected were hard, white, and dense: when they were divided, the appearance of the cut surface was the same.

The smaller nodules upon the skin were hard and firm: they had the colour of the skin, which seemed thickened and hardened, and had become distinctly laminated, to form them. The larger nodules differed from the rest in this, that the cellular membrane beneath them was converted into a like substance, a thin layer of fat partially intervening between it and them. On lifting off the crust from the largest, the surface was found to be soft and vascular, with something of a villous appearance.

The structure which I have described as met with in the gland of the right breast in the preceding case, is the common appearance of scirrhus.

I removed, by the advice of Mr. Cartwright, the breast of a lady, on the 2d of May, in the present year. The lady's age is sixty-eight. The tumor had been forming two years. There had been some doubt about its nature, as it had not been attended with lancinating pains, and as there was an evident collection of fluid in it immediately to the outside of the nipple. But elsewhere the tumor felt like a scirrhus: it was of the size of an egg. The nipple was flat, but not retracted. The lymphatic glands were not affected.

On examining the tumor after its removal, its substance was exactly what I have described above. The fluid, which had been felt, was contained in a cavity of the scirrhous substance: it amounted to about two ounces, and was partly serous, partly viscid, of a dirty yellow colour.

The lady continues perfectly well at the present time; and her age and the slow growth of the scirrhus being considered, it is probable that the disease will not return.

On the 3d of October, I removed the breast of a lady, whom I attended with Mr. Allan, of Epsom. Her age is forty-three. She is married, and has had twelve children. A year ago, a hard lump formed in the axilla, near the outer margin of the right breast, which was taken out by an operation last February. It is her impression, and very possibly an erroneous one, that at that time the breast itself was affected.

When she placed herself under my care, in September last, the cicatrix of the former operation adhered to a tumor below, which felt like an enlarged lymphatic gland; and the neighbouring corner of the breast had the characteristic induration of scirrhus. Three fourths of the breast seemed healthy; the nipple was not retracted. She suffered lancinating pains in the hardened part. I removed the whole breast, with the cicatrix, and two indurated glands and some thickened membrane.

The indurated portion of the breast had the character described above; the glands and hardened membrane were white and dense.

The following case hardly, perhaps, deserves a place among instances of scirrhus, but its partial resemblance to the preceding leads me to mention it.

Ann Shaw, ætat. twenty-six, unmarried, was admitted into the Middlesex Hospital, July 29th, 1828, with a small hard tumor, immediately below and to the outside of the right breast. The tumor, which was then of the size of the last joint of a finger, had been first observed four months before: it was then smaller, and had gradually enlarged to its present size. It was in some degree tender on pressure, and was attended with shooting pains. Leeches and fomentations, and an alterative course of medicine, were used during three or four weeks, without advantage. I therefore thought it prudent to remove the little tumor, which I did a few days after the patient came into the hospital. The tumor appeared to consist of an enlarged and hard lymphatic gland. The wound healed readily. Some shooting occurred in the cicatrix a few weeks afterwards, but it went away upon the application of leeches and warm fomentations; and I have every reason to believe this patient has continued, and continues, quite well up to the present time.

Mary Dale, ætat. fifty-eight, came from the neighbourhood of Dorking, to be admitted into the Middlesex hospital, on the 19th of March, 1828, with a hard tumor of the left breast, of the size of a moderately large walnut. The tumor had been first observed two years and a half before her admission. During the last four months, it had been the seat of pricking, shooting, and lancinating pains. During the last month, sensations of a similar description had been felt in the left axilla, where, upon examination, two glands were found enlarged to the size of a hazelnut, not very hard, but extremely tender on pressure, which the breast was not.

Under a strong impression that the breast was affected with scirrhus, and that the glands felt in the axilla were merely enlarged from inflammatory irritation, I amputated the former, and left the latter; the removal of which would have rendered the operation considerably more severe.

I should not, however, in similar circumstances, again leave enlarged glands near a scirrhus; which the tumor in the breast in this instance was supposed, and proved to be. Nevertheless, I have not learnt that, in the case of Mary Dale, this practice has been unsuccessful. The suspected glands, indeed, swelled considerably within a short time after the operation, during the healing of the wound; but, on the repeated application of leeches and poultices, they subsided, and before she left the hospital, where she remained several months, they were scarcely larger, or more distinctly to be felt, and were not of a harder substance, than the glands of the opposite axilla. It is now nearly a year since I have seen or heard of this patient.

### II.

A lady, ætat. sixty-six, was attended by Mr. North for a small tumor in the inner and upper part of the left breast.

She had been aware of its existence about seven months. It was slightly tender when pressed upon; it was not hard, but felt like common inflammatory thickening of a lobe of the breast. In July last, Sir Astley Cooper saw this patient with Mr. North: he entertained the same view of it which Mr. North had taken, and prescribed a mercurial alterative at night, with a bitter and aperient draught in the morning; the part to be bathed with a spirit lotion. This plan was pursued for a few weeks, during which the complaint continued stationary.

In August I saw this patient with Mr. North: the part had become somewhat painful; it had slightly increased in size, and there was fulness of the glands of the axilla; but the character of the tumor had not altered. We agreed that leeches should be occasionally applied, and the mercurial alterative used again. The symptoms yielded to this treatment; the breast ceased to be painful.

The lady now left town: on her return, the beginning of October, the symptoms had greatly changed for the worse. The tumor of the breast, although not much larger than before, had now all the character of scirrhus; at the same time, subcutaneous tumors had begun to form in different parts of the body. Her breathing became affected; she was unable to lie down in bed. She then sunk rapidly, and died on the 1st of November.

The body was examined by Mr. North and myself, with Mr. Arnott. The lump upon the inner edge of the left breast was prominent, so as to raise the integuments: it had to the touch the consistence of a scirrhus. Two other parts of the breast felt indurated, but not in the same degree. On making a section of the gland, the lump at the inner edge presented the following appearance: it was nearly spherical; its substance was particularly hard and dense, slightly elastic, and mottled grey and white, the white predominating; the margin was here and there of a softer texture, and, for the depth of a third of an inch, highly vascular, or bloodshot. The morbid structure ended abruptly.

The other two indurated parts had a different character: the induration began insensibly, so that it was difficult to determine exactly where the natural structure of the breast ended. The central part of each was dense, slightly elastic, of a dull white colour; and, in one of the indurations alone, the white was partially mottled with grey. In the condensed substance at one part, and at another in the soft and natural substance of the breast, there was a body, of the size of a pea, that was internally soft and bloodshot, and resembled, in some degree, the margin of the lump first described.

The right breast had partaken of the same affection: it was indurated in two places, so that, deducting from the preceding description of the left breast the account of the more advanced and maturer tumor, the rest will stand as a sufficiently exact account of the state of the right.

The tumors in the subcutaneous cellular membrane were about as large as middle-sized peas: there were from twenty to thirty dispersed about the abdomen, immediately below the integuments, to which they did not adhere; a considerable group was situated below the right breast, and there were a few, individually of larger size, about each clavicle; and at least a dozen upon the back, between the shoulders. Almost all these tubercles had one character: when entire, they felt firm and tense; when cut through, a quantity of fluid escaped, in some serous, in others thicker and whitish. The parenchyma of the tumors was a soft, grey, spongy flesh, highly vascular or bloodshot, and exactly resembling the pealike tumors in the substance of the breast. Two or three only of the little tumors about the right breast, when cut through, were of that dense white substance, which is the commonest state of the glands about a scirrhous breast.

It appeared to Mr. Arnott, Mr. North, and myself, that all the little subcutaneous tumors were diseased lymphatic glands.*

In the substance of each lung, and in the liver, there were several tubercles, of dense, white, elastic substance.

A poor woman, thirty-eight years of age, who had borne a family, was admitted into the Middlesex hospital in March last, with disease of the left breast. Six months before, she had observed a hard lump in the gland, the whole of which, at the time of her admission, was affected, and had the characteristic induration of scirrhus; the nipple was retracted. Two or three glands in the axilla were enlarged, but they appeared quite within reach.

* I need not remark that minute subcutaneous lymphatic glands are extremely numerous upon the trunk, head, and neck, although they seldom form a feature in disease. I recollect some years ago being sent for, to see a young gentleman, of a strumous diathesis, who had been exposed to cold and damp, and had a slight attack of fever, with a sudden growth of twenty to thirty little painful and exquisitely tender nodules, as big as peas, about the back of the head and neck, which almost prevented him laying his head on his pillow. I treated the complaint as a severe cold, and in two or three days the swelled glands were much less painful, and had begun to subside.

The breast, together with the glands, was removed on the 6th of March. The breast had the true character of scirrhus, except at the outer and lower part, where the surface of the diseased structure was softer, very vascular, with a cavity containing a dark red serum.

Five glands were removed. One, a flattened sphere an inch in diameter, was filled with a firm, whitish, caseous substance, which admitted of being squeezed out in coarse granules. The smaller of the diseased glands had each a large nucleus of the same substance.

On the fourth day some fever supervened; the parts became swollen and painful. One or two strips of adhesive plaster were reapplied on changing the dressings, with a poultice over the whole. The skin was not red, but looked, with the membrane below, thick and swollen.

In two or three days a profuse discharge came from the interior of the wound: it was sanious, with a large quantity of oil floating in it. The wound now went on favorably, and healed. But, in six weeks from the operation, the parts about the cicatrix began to thicken, and enlarged rapidly into an immense mass of fungus, which occupied the whole of the left side of the chest and axilla, and part of the right side. This broke, and sloughed rapidly, being attended with great pain; and the poor patient sank, and died within five months after the operation.

She found some alleviation of her suffering in the application of folds of linen, steeped in an aqueous solution of opium, to the open and sloughing surface.

In October 1827, I removed the right breast of a lady, ætat. fifty-seven, whom I attended with Mr. Cartwright. She was of a full habit, and the breast was large, from the quantity of adipose substance. The whole gland was uniformly scirrhous. The disease had been forming several months. No lymphatic gland was enlarged; but the cellular membrane was observed to be generally, though very slightly, indurated. Little hard points were felt on pressing portions of the adipose substance that had been removed in the operation.

The wound healed favorably; but in five months the parts round the cicatrix began to enlarge, and in the course of a short time the whole axilla and right side were occupied by a large, rounded, and lobulated mass, of the consistence of firm dough. This lady survived the operation a year and a half. The fungous tumor broke, and began to slough, a short time only before her death.

The fungoid growth, which often supervenes in scirrhus of the breast, commonly resembles in texture the firmer part of a fungus hæmatodes. Occasionally it partially retains something of that fibro-cartilaginous texture which belongs to scirrhus.

Pure fungus hæmatodes is comparatively an infrequent disease of the breast. I witnessed the following case of that complaint, when house surgeon to the Middlesex hospital.

A young unmarried woman, ætat. nineteen, was admitted into the hospital, under the care of Mr. Cartwright, in March 1819. Six years before, a tumor, unattended with pain, formed under the right breast: the whole of the breast gradually became involved in the disease. In the course of another year the left breast became affected. At the time of her admission both breasts were considerably enlarged; the right, however, was a third larger than the left. The colour of the left was natural; that of the right dark, inclining to the colour of old mahogany. Both breasts were to the touch firm, with a consistence like dough. The surface between the breasts was hard and prominent; the skin thickened and nodular. At this part there were two ulcerated holes, an inch in diameter: the surface of the ulcers was dark. This patient had not experienced much pain: that which she had felt had been rather smarting than pain, and had been confined to the right breast and to the ulcerated part. She died soon after her admission.

The left breast (the smaller) was of a hard, white, firm, and fibrous, but doughy substance; here and there a calcareous concretion was found in it; in parts the texture was softer, more like the substance of brain, and slightly bloodshot in parts.

The right breast was generally softer, and great part of it consisted of brainlike substance, here and there streaked with blood.

The substance between the breasts, and the lymphatic glands, were white and firm; the white, doughy substance admitted of being squeezed out in a caseous or pulpy consistence.

The right pectoral muscle was pale and wasted. The interstices between the fasciculi were studded with numerous small tubercles, which, when squeezed, yielded a white pulpy substance. This white, doughy, or pulpy formation was found both in the pleura costalis and the pleura pulmonalis, on the right side. There was a large quantity of water in the cavity of the right pleura.

III.

A lady, fifty-eight years of age, married, but never having borne children, observed for the first time about six years ago, a hardness at one part of the right breast. About two years after this, she had a fever, during which the tumor disappeared. In 1827 the tumor returned, but no remedies were made use of till March 1828, when she placed herself under my care. Mr. Brodie saw her at that time, in consultation with me.

The right breast was enlarged, and formed a flat oval cake, five inches in length, four in breadth, and about two in thickness. To the touch, the tumor was firm, and slightly elastic, its surface even, or very slightly lobulated. It was easily moveable upon the pectoral muscle. The nipple was slightly retracted. The skin over the tumor was not tense; the subcutaneous veins were enlarged. At times shooting pains were felt in the breast, and there were two points at which the tumor was tender on pressure: one internal to the nipple, the other at the upper and outer part. This lady is of a pale complexion and relaxed habit, liable to occasional partial attacks of erysipelas upon the face, and continually requiring small doses of aperient medicine to move the bowels.

℞. A drachm of Liquor Potassæ to be taken three times a day in table-beer. A drachm of the ointment of hydriodate of potass to be applied to the tumor night and morning.

The adoption of this plan was of temporary service. At the expiration of two months, the general health had greatly improved; the bowels no longer needed medicine; the shooting pains in the breast had entirely gone away. The tumor had not enlarged, and had become less tender on pressure.

The lady now went into the country, with directions to continue taking the Liquor Potassæ, in a dose increased to a drachm and a half three times a day.

Towards the winter this lady returned to London, having steadily pursued the plan laid down, in all but the constant use of the ointment, which at one time irritated the skin. About the middle of December 1828, the changes that had taken place were the following: The breast had enlarged considerably; the shooting pains had recurred; and there was some tenderness in the axilla, but no enlarged gland could be felt there. I directed the application of leeches to the axilla, with hot fomentation afterwards, and the tenderness in the axilla went away.

It appeared to me that no time was to be lost in removing the breast. Sir Astley Cooper, who saw this case in consultation with me, concurred in this opinion. I amputated the breast early in January last.

Upon examining the part after its removal, the disease, it appeared, had not involved the whole breast, part of which lay compressed and condensed to the right of the tumor, towards the axilla. The nipple adhered partly to the unchanged breast, partly to the tumor, but was readily and cleanly separable from the adjoining parts. The tumor was about three inches in thickness, five in length, four in breadth. It was weighty, and tense and distended. On cutting it through, the texture generally had a crispness like unripe fruit: the surface of the section was of a light grey colour, glistening, and semitransparent; the interior texture succulent, in the softest parts tearing, when a little force was used, into gelatinous strings; at the circumference, the texture became more dense, and approached more nearly, in colour and consistence, the character of true scirrhus. There was no cavity in any part of the tumor.

On the fourth day after the operation, the patient complained of pain at the lower and inner angle of the wound. The wound was dressed: adhesion seemed to have taken place along the greater part, and there was no inflammatory tumefaction, or unusual tenderness, where the uneasiness was felt; yet, when she drew in her breath, there was some pain at this point, which caught her, and suddenly checked the inspiration. This symptom had come on during the preceding night. The pulse was ninety; the skin something heated, but rather moist than otherwise; the tongue neither furred nor dry. I thought it right, under these circumstances, to take away some blood: when she had lost fourteen ounces she became faint, and said that the pain had nearly left her. The blood showed that it would have a size. In less than an hour the pain recurred more severely than before. She was again bled to sixteen ounces, when she became faint again, and the pain was scarcely felt. She then fell into a dose, and, on awaking, declared herself entirely relieved from the pain and oppression of breathing.

Every thing now went on favorably, and the wound had healed in a fortnight after the operation. The lady is now in perfect health.

I am induced to add some illustrations of open cancer of the breast, or of scirrhus which has gone into ulceration.

With this view I select two cases at present under my care in the Middlesex hospital; the first an instance of simple cancer, the second of fungoid cancer.

Esther Christie, ætat. sixty-five, (Whitbread's ward;) a widow, has borne children. Four years ago a tumor formed in her right breast, which, in a year, began to ulcerate. She has now been two years in the hospital, under my care. When she was admitted, there was an extensive ulcer upon the right side of the chest. The character of this ulcer has not materially changed during the last two years; but, in point of size, it is a fourth larger than at the time of her admission: its area is rather more than sixteen square inches. The surface of the ulcer is tolerably even; it is covered with large, firm, red granulations; towards the middle there is a strip of surface which is imperfectly cicatrised. The edges of the ulcer are elevated; they are but little discoloured, and of the true hardness and touch of scirrhus. This hardness extends to a very small distance from the edge of the ulcer. The edge of the ulcer is very irregular, here inverted, there everted, as it has happened that the ulcer had eaten away in different parts more or less of the scirrhous substance round it. It is exceedingly remarkable that there is not the most trifling glandular enlargement in the axilla or about the clavicle of this patient: she cannot, however, raise her right arm to any height. The pectoral muscle in the axilla feels hard and rigid; there is pain down the outside of the arm, from the shoulder to the elbow.

At times this patient suffers violently with acute and darting pain; at other times she suffers little. During an exacerbation of pain, one or other edge of the ulcer, which for the time is the seat of pain, is tumid, and the adjacent ulcerated surface itself of a bright and angry red. On these occasions the application of eight or ten leeches, at a little distance from the edge of the ulcer, has generally proved beneficial.

She has tried every variety of local application: that which she commonly uses is simple ointment; that which has given her, when suffering, the most marked relief, is a mixture of the Liquor Plumbi dilutus with an aqueous solution of opium, or else the aqueous solution of opium alone, applied either as a lotion or as the liquid part of a poultice. A lotion consisting of a drachm of the prussic acid of Scheele to a pint of distilled water, appeared to her to irritate the ulcer; the same reduced to half its strength was nugatory in its effects.

In several cases of cancer of the uterus, I have found a solution of a drachm of prussic acid in a pint of distilled water extremely beneficial, as an injection, in soothing pain. When the pain is extremely severe in such a case, I have known the abstraction of blood by cupping upon the sacrum, or, in a patient debilitated by profuse hemorrhage, the temporary application of cupping glasses without scarifying, produce immediate relief.

In connexion with the case of Esther Christie, I may mention that of a patient who died three months since in the hospital, where she had been an inmate nearly as many years. Five years ago, her right breast had been removed by Mr. White. Two years after, the disease returned in the cicatrix. It was singularly slow in its progress. The cicatrix lay a narrow hollow between the edges of the skin, which were thickened, hard, and irregular; the pectoral muscle was indurated towards the axilla, and two or three small, hard glands adhered to its axillary surface. This patient could not raise her arm to any distance from the side: the arm was not swollen, but she experienced pain about the insertion of the deltoid, and the elbow-joint was always in some degree contracted: it was frequently fixed at a semiflexion, with the tendon of the biceps as rigid as a cord. At such a time, the attempt to extend the elbow, or even rudely touching the arm, gave great pain. Nothing relieved this symptom so much as the application of large, hot linseed poultices around the elbow-joint.

The cicatrix had scarcely begun to ulcerate, when this patient died, exhausted by long suffering.

Elizabeth Benson, ætat. sixty, was admitted into the Cancer ward within this month. Her right breast, which is alone affected, presents a well-marked instance of fungoid cancer. The disease began two years ago, as a small tumor of the lower part of the breast; for six months it gave no pain: it broke into an open ulcer about twelve months ago.

In her the ulcer appears deep, from the height of the surrounding fungoid growth, much of which has formed since the disease became an open cancer; the surface of the ulcer is covered with granulations. From one part a portion of the tumor has recently separated by sloughing. The part of the tumor which remains is tender upon pressure; its colour a deep red, mottled with circular patches about the size of a sixpence, which are prominent and white, from the fungous growth which is here pushing up the integument; its texture feels hard and firm.

This patient finds some relief from the application of a bread poultice, with a tablespoonful of the weak prussic-acid lotion stirred into it. The same quantity of the stronger lotion increased the smarting pain which she suffers in the breast.

In descriptions of scirrhus and cancer of the breast, the details terminate either with the performance of a painful operation, or in the history of protracted suffering from the ravages of disease. If the part which has been attacked cannot be removed by an operation, the care of the surgeon is limited to sooth and to lessen the severity of continually increasing bodily pain.

It is evident that the first point to be aimed at in the study of such a disease, is to learn to determine what are the cases in which the affected parts may be removed, with a fair probability of eradicating the complaint.

From the little experience which I have had, I am inclined to suppose that, where the disease is confined to the breast, where the tumor is small and has been slow in its growth, there is reason to expect that the operation will prove entirely successful. I believe, likewise, that the chance of success is greater early and late in life, than at the middle period of life.

Where the tumor is large, where it has grown rapidly, where the whole of the parts engaged cannot be removed, where the skin is diseased over the indurated breast, there the operation for scirrhus is unavailing, and only tends to produce a more rapid and painful form of disease than that which it temporarily removes.

19, *George street, Hanover square;*

*December* 13*th*, 1829.

